# Sexual Torture in Palestinian Male Detainees: Epidemiology, Impacts and Outcomes

**DOI:** 10.3390/healthcare14081105

**Published:** 2026-04-20

**Authors:** Mahmud Sehwail, Khader Rasras, Wisam Sehwail, Pau Pérez-Sales, Andrea Galan-Santamarina, Raluca Cosmina Budian

**Affiliations:** 1 Treatment and Rehabilitation Center for Victims of Torture (TRC), Ramallah P606, Palestine; sehwail@trc-pal.org (M.S.); wisam.sehwail@trc-pal.org (W.S.); 2 SiR[a] Center, C. de Pinos Baja, 41, Tetuán, 28029 Madrid, Spain; pauperez@runbox.com (P.P.-S.); andrea.galan.sta@gmail.com (A.G.-S.); 3 Community Action Group (GAC), C. de Pinos Baja, 41, Tetuán, 28029 Madrid, Spain; 4 Esade Business School, Ramón Llul University, Av. Torre Blanca, 59, Sant Cugat, 08172 Barcelona, Spain

**Keywords:** sexual torture, sexual violence, ill-treatment, Israel, Palestinian detainees

## Abstract

**Background**: Torture, as a fundamental violation of human rights, is unequivocally condemned by all international organizations. Sexual torture is one of the most severe forms of torture, encompassing forced nudity, various forms of humiliation, and physical abuse, including rape. Despite testimonial evidence indicating the incidental use of sexual torture by Israeli authorities, there is a lack of epidemiological research providing a comprehensive understanding of this issue. This study aims to analyze the prevalence and characteristics of ill treatment and sexual torture among Palestinian male detainees and the subsequent impacts. **Methods**: This cross-sectional study analyzed a database of 517 former male detainees. The interview protocol included items related to psychological and physical methods of sexual torture, medical impacts, subjective psychological impacts, clinical medical and psychological measures, and psychosocial and community impacts. **Results**: The findings indicate that the majority of detainees experienced some form of sexual torture, with humiliation being the most common type. The impact of sexual torture are severe, affecting both clinical and social domains. The impacts of sexual torture persist over time and, in some cases, worsen, particularly regarding physical health outcomes. Socially, the consequences extend to the detainees’ families and communities. **Conclusions**: The prevalence of such torture tactics calls for urgent responses from both the authorities and civil society. These findings highlight the need for proactive measures to address and mitigate the impacts of sexual torture, including independent investigations, robust monitoring, secure reporting mechanisms, the prosecution of perpetrators and comprehensive reparation for victims.

## 1. Introduction

In the context of the Israeli–Palestinian conflict, the detention of hundreds of children, young people, and adults across genders is documented every year [1,2,3,4]. According to Israeli human rights organization HaMoked, in September 2023 approximately 9000 individuals classified as “security prisoners” were held in Israeli custody, including 3484 administrative detainees and 606 individuals designated as unlawful combatants. As of 31 December 2023, administrative detention included 49 minors and 8 women, while the category of unlawful combatants comprised 11 minors (10 boys and 1 girl) and 42 adult women. Additionally, Israel holds 899 individuals classified as “unlawful combatants” [1]. There are numerous testimonies and data on the widespread use of torture in the context of such detentions [5].

Torture is a violation of human rights and a crime under international law, unequivocally prohibited and unjustifiable under any circumstances. Sexual torture is one of the most severe forms of torture [6]. In contexts of violence around the world, sexual violence has always been present as a means of furthering military and political objectives [4,7,8]. Sexual violence is also used as a weapon of war because it attacks the identity of the detainee and has profound impacts on the person, their family, and the community.

The World Health Organization (WHO) defines sexual violence as “… *any sexual act, attempt to obtain a sexual act, unwanted sexual comments or advances, or acts to traffic, or otherwise directed, against a person’s sexuality using coercion, by any person regardless of their relationship to the victim, in any setting, including but not limited to home and work*” [9]. It also defines sexual torture in the context of detention.

Different forms of sexual torture have been documented in almost all active conflicts [10,11,12,13,14]. Sexual harassment is defined as inappropriate behavior of a sexual nature, regardless of its purpose. It may be a form of intimidation, exercise of violence, or simply abuse of a position of power for no explicit purpose using sexual-related means [15,16,17,18]. According to its legal definition, rape is defined as a sexual assault consisting of vaginal, anal, or oral carnal access by the introduction of bodily members or objects [19]. Sexual torture in men has been considered systematically underestimated while its consequences are at least as severe as in women [4,20,21,22,23,24]. Also, the Oxford English Dictionary defines sexual harassment as “Harassment (typically of a woman by a man) in a workplace or other professional or social situation, involving the making of unwanted sexual advances, obscene remarks, etc.”.

The full extent of sexual torture against men is currently unknown, although it is likely to be more prevalent than reports suggest due to under-reporting and widespread stigma surrounding it [17]. In the literature, the lack of up-to-date data on sexual torture is largely attributed to the absence of institutional detention, insufficient legal frameworks, and a narrow focus on sexual violence. This issue is exacerbated by under-reporting, unrecognition, and under-representation due to a lack of awareness and advocacy, particularly [13].

Overall, acts of sexual violence perpetrated against men in times of conflict have an impact not only on the individual directly affected but also on his family and possibly the entire community [4]. From a broader perspective, when instances of sexual violence are perpetrated against Arab men by those outside the Arab cultural sphere, it can be interpreted as an attempt to impose foreign customs and disrupt existing societal norms associated with the concept of masculinity in Arab culture [13,18,25]. Sexual torture and ill treatment, including forced exposure and verbal abuse with sexual connotations, can cause particularly deep and sometimes enduring feelings of shame among Arab men [2]. This is rooted in the concept of dignity and honor, a fundamental aspect of societal dynamics prevailing in a significant part of the Muslim sphere [25,26]. Sexual torture seeks to dismantle established social norms related to sexual identity and difference [27] and break basic beliefs and identity [28]. Therefore, it is essential to take into account social and cultural factors such as family, community, gender, and religion when assessing sexual torture and/or ill treatment as these elements play a significant role in shaping the impact and response to such violations.

In the context of the armed conflict in Palestine since the establishment of the State of Israel in May 1948 [29,30], some disturbing data suggests the use of sexual torture on Palestinian detainees, through reports from national and international organizations [28,31,32,33,34]. There is only one academic study published to date that analyses the prevalence of sexual torture in the database of beneficiaries of the Israeli organization Public Committee Against Torture in Israel (PCATI). The study concludes that Israeli interrogation and/or law enforcement authorities were involved in torture and ill treatment of a sexual nature of Palestinian men and boys between 2005 and 2012. The victims were aged between 15 and 43 years at the time of the incidents (median age: 24). Most victims (N = 26; 43%) were between 20 and 29 years old; nine (15%) were minors; and six (10%) were aged 18–19. Eleven victims (18%) were aged 30–39, two (3%) were older, and age information was unavailable for six victims (10%) [2]. Also, in the study, more than 1500 testimonies and forensic reports from the PCATI team were screened [2]. Of these, 77 testimonies (about 4%) indicated incidents of sexual torture or ill treatment against Palestinian boys and men. The study collected 36 reports of verbal sexual harassment and 35 reports of forced nudity. Moreover, there were six testimonies of Israeli officials involved in the physical sexual assault of arrested or imprisoned Palestinian men. Physical assault in most cases involved pressing and/or kicking the genitals, while one testimony involved a simulated rape, and another described an actual rape with a blunt object. Most allegations of sexual torture or ill treatment concerned situations during arrest by either soldiers or border police officers and during interrogation. Although not rare, sexual torture allegations during police arrest or detention either at police stations or in jail were less frequent. In their conclusions, the authors stated that there is a need for more detailed and systematic documentation and codification of data.

A study based on the testimonies of 121 Palestinians detained in the Israel Security Agency (ISA) Petah Tikva interrogation facility in the first and last quarters of 2009, some for up to two months, found that 43 (35%) reported that interrogators addressed them with swear words and degrading language of a sexual or humiliating nature and in particular with insults of a sexual nature about women in the family; 68 (56%) detainees reported that interrogators threatened them. The most serious examples included threats to keep the detainee in isolation for many months and sexual threats [33].

The Al-Mezan Center conducted a study with 107 Palestinians detained in Gaza by the Israeli Security Forces between 1 November 2013 and 31 October 2014. They reported repeated beatings to sensitive parts of the body in 27% of cases, being stripped naked in 9%, and threats to rape relatives in 1% of cases [35].

Anecdotical testimonies of sexual harassment against males are also described in other reports [36,37,38,39]:

*“As they carried him, blows from soldiers and sexually degrading insults against Mohammad’s mother and sisters continued to rain down (…) deliberately exploit Palestinian society norms where women are traditionally given more protection and special status, while the preservation of their honour is of the utmost importance”.*

*“They took me out for interrogation for about 12 h, and then I spent another 12 h in the refrigerator. They gave me three meals that were okay. The interrogators hit me with their hands all over my body. Two held me down and the rest hit me. They threatened me with sexual assault but didn’t follow through on it”.*

[35]

There are also reports of torture and ill treatment of children to extract forced confessions. Most disturbing is the prevalence of sexual harassment and sexual abuse of Palestinian children in Israeli detention centers. In 2010, Defence for Children International—Palestine Section (DCI-Palestine) described the use of sexual harassment to obtain confessions as “widespread and systematic” [40,41]. It estimates that almost every affidavit signed by child detainees contains some aspect of sexual harassment or sexual abuse during the interrogation phase of detention [34].

In 1999, the High Court of Justice (HCJ) banned the use of specific torture methods, including shaking, the frog crouch, and the shabeh method. But Israel’s Supreme Court ruled, at the same time, that while torture and other ill treatment were generally prohibited, Shin Bet interrogators who used what the Court described as “physical interrogation methods” in “ticking time bomb situations may be exempted from criminal prosecution or even investigation. (The ticking time bomb scenario is a hypothetical ethical and legal dilemma used to justify the use of torture in extreme situations. It posits that authorities have captured a terrorist who has knowledge of an imminent attack (e.g., a bomb set to explode in a crowded area), and the only way to prevent mass casualties is to extract the information through coercive means, including torture. This scenario is often debated in discussions on human rights, counterterrorism, and legal frameworks, with critics arguing that it presents a highly unrealistic and subjective justification for state-sanctioned torture.) In other words, torture was allowed in the so-called “ticking time bomb”, without any need for further justification. Therefore, this ruling has been controversial and criticized by human rights organizations and the United Nations Committee Against Torture. Consequently, it is essential to analyze the impact of these landmark sentences and whether allegations of torture diminished after 1999 [1].

Our study analyses the database of the Ramallah Treatment and Rehabilitation Center for Victims of Torture (TRC) with data from 1987 to 2018, which allows for a historical analysis of sexual torture practices. The center was founded in 1997 and is part of the International Rehabilitation Council for Torture Victims (IRCT), a Denmark-based network of 160 member centers across 76 countries. These centers provide essential services, including medical and psychological rehabilitation, to survivors of torture and advocate for their human rights and justice, providing services to more than 60,000 survivors worldwide.

The TRC provides care for victims of political violence and their families in the West Bank. Throughout its existence, it has maintained an extensive clinical database and carried out several important epidemiological studies, the data from which has been used to plan rehabilitation activities [42,43,44,45,46,47,48]. The present study systematizes data on sexual torture.

Therefore, the primary objectives of this research are delineated as follows: (1) to examine the prevalence and characteristics of sexual torture and ill treatment experienced by Palestinian men in detention, situating these acts withing the broader context of torture perpetrated by the Israeli authorities; (2) to assess variations in these practices before and after the 1999 Israeli Supreme Court sentence regarding the use of torture in detention; and (3) to analyze the clinical, medical, and psychosocial impacts of sexual torture on detainees, including their temporal evolution.

## 2. Materials and Methods

*Definition of sexual torture in the context of detention:* For this study, sexual torture was considered when the person informed one or more of (a) threats related to sexual integrity, i.e., sexual threats and the witnessing of others being sexually harassed or abused; (b) humiliating and degrading acts against sexual integrity, i.e., forced nakedness and sexual humiliations; and (c) physical sexual assault, i.e., sexual acts involving direct physical contact between victims and torturer, between victim and victim, between victim and animal, or all of the above, as well as violence against the sexual organs, i.e., the introduction of objects or beatings or kicks in the genital parts.

*Sample selection and data collection processes:* Since its establishment in 1997, the Treatment and Rehabilitation Center for Victims of Torture (TRC) has developed a comprehensive database of individuals released from Israeli detention, based on official records from the Ministry of Detainee and Ex-Detainee Affairs of Israel, and individuals affected by broader human rights violations, including family members and others seeking support, within the framework of the accompaniment and rehabilitation process.

This cross-sectional study drew exclusively on the 600 most recent cases of former detainees recorded in the TRC database. After excluding cases with missing data and restricting the sample to males, the final analytic sample comprised 517 participants (age range: 16–61 years; M = 31.5, SD = 9.44), with an average detention duration of 19.3 months (SD = 27.39). No new participants were recruited, and a post hoc power analysis was conducted assuming an effect size (Cohen’s d) = 0.50 and α (Type I error) = 0.05, which yielded power = 0.99 to detect effects of that magnitude. This selection may introduce a degree of selection bias, which is acknowledged as a limitation of the study.

Data was collected through structured home-based interviews conducted by trained TRC therapists between June and July 2019. All data reflects retrospective accounts of detention experiences that occurred between 1987 and 2018, and the analysis was anchored to the detention episode identified by each participant as the most significant in terms of personal impact. Participants were primarily contacted through direct outreach by phone. Additionally, some consenting respondents helped identify other released detainees using a snowball sampling approach. However, peer referral accounted for less than 10% of the total sample, ensuring that most cases were identified through the official list of people released from Prison.

### Measures

1. The TRC Checklist of psychological and physical coercive methods, a 140-item checklist grouped into 22 categories, was used in daily clinical work in the TRC center. Each item is ranked on a 4-item Likert scale: never happened, happened once, happened twice, and happened three or more times.
2. Clinical measures included (a) the Arabic version of the Beck Depression Inventory (BDI-II) [49], a 21-item inventory that measures the severity of self-reported depression over the last two weeks, and (b) the Post-Traumatic Stress Disorder Inventory (PTSDI), a list of 20 symptoms of PTSD that follow DSM-V Revised criteria based on the Arabic version of PCL-5 [50], adapted for the Palestinian population by mental health experts who pilot-tested it with a small cohort to confirm content validity, clarity, relevance, and contextual acceptability. Although challenging field conditions precluded a full psychometric validation process, our expert-driven approach ensures that the scale retains its core validity and accurately captures clinically meaningful symptom levels.
3. Psychosocial measures included (a) the Family Impact Scale (FIS), a 7-item measure of the impact of detention on the role of the detainee in the family, and (b) the Social and Community Impact (SCI), a 5-item measure on the impact of detention on labor and social functioning. All items are dichotomous.
4. Medical impact was assessed through an 8-item checklist of medical consequences of torture, with dichotomous answers.

The psychosocial and medical measures are based on a questionnaire carried out by the TRC itself, based on the impacts reported in the United Nation’s (UN) Manual on the Effective Investigation and Documentation of Torture and Other Cruel, Inhuman or Degrading Treatment or Punishment, also known as the Istanbul Protocol [51].

The TRC Checklist of Coercive Methods, the Family Impact Scale (FIS), the Social and Community Impact (SCI), and the Medical Impact Checklist were created specifically for comprehensive documentation of survivors’ experiences. Items were derived from a literature review, developed by context-knowledgeable experts, and piloted with Palestinians released from Israeli prisons. These tools were not intended as stand-alone psychometric instruments and therefore did not undergo formal validation procedures. Instead, their strength lies in a pragmatic, context-sensitive design that reliably captures the breadth of traumatic exposures and psychosocial impacts, while the adapted clinical measures cover core symptom domains.

*Statistical Analysis:* We used cross-tabulation, with chi-square statistics for frequencies and a *t*-test to determine the difference between means. First, a descriptive analysis of sociodemographic variables and methods of sexual torture was conducted, using cross-tabulation tables to explore the prevalence and characteristics of sexual torture methods, as outlined in the first objective. To examine the evolution of sexual torture over time, a comparison was made between pre and post 1999 practices using again cross-tabulation tables. Finally, *t*-tests and chi-square tests were used to compare the impacts faced by survivors who had experienced sexual torture with those who had not, as well to examine the evolution of these impacts over time among sexual torture survivors. This addresses the second and third objectives, providing insights into the specific consequences faced by survivors. All analyses were conducted using SPSS (23rd version) during 2022 and 2023.

*Ethical aspects:* Participants were identified within the framework of the accompaniment and rehabilitation process. Semi-structured tools and questionnaires were used to assess impacts and guide therapeutic planning. Additionally, authorization was obtained for the subsequent use of this data for research purposes. Thus, participants’ consent for the inclusion of their data in this article was explicitly granted. The Ethical Committee of the TRC Center and the Ethics Committee Board of the University of Babes-Bolyai (STAR-UBB) approved the study (8218/16.07.2021). The study complies with the Ethical standards and procedures for research with human beings of the World Health Organization [2].

*Author reflexivity*. Our research team is composed of scholars and practitioners dedicated to the study of human rights. It includes researchers of Palestinian origin who conduct their work in the region, alongside European collaborators who support their efforts. The team comprises both men and women with varying levels of experience, including senior researchers with extensive expertise in the field as well as mid-career scholars. This diversity in geographic background, gender, and professional seniority enhances the rigor and scope of our research, ensuring a critical and contextually grounded approach to the study of human rights in highly vulnerable settings.

## 3. Results

### 3.1. Prevalence of Sexual Torture

Table 1 shows the prevalence of sexual torture among Palestinian male detainees. In men, more than 80% of cases involved some form of sexual torture. The overall prevalence of sexual torture, without differentiation by type of sexual violence, appears to be consistent regardless of age, detention region, or year of detention.

Analyzing the different types of sexual torture, we found that the most prevalent form is humiliating and degrading acts against sexual integrity (68.1%).

At the time of the study (before the war in Gaza from October 2023) there were higher levels of humiliating and degrading acts against sexual integrity in the case of the West Bank compared to the Gaza Strip. At the same time, physical sexual assault was more common in the Gaza Strip than in the West Bank.

Analyzing types of sexual torture in men (see Table 2), forced nudity is the most common form of sexual violence (60.9%), followed by beating on the genitals while partially undressed (58%), mockery of masculinity (35%), and mockery of the body while undressed (28.2%). Other forms of sexual torture include threatening to rape female family members (10%), threatening to rape or sexually assault the detainee (9%) and bringing in partially undressed female soldiers and revealing significant parts of their body, beyond military regulations, to provoke handcuffed detainees to cause them to have a psychological breakdown (7%). In 3% of cases, there was a sexual assault, including touching without rape. None of the detainees reported rape.

A slight decline in the prevalence of sexual torture methods is observed after 1999. Physical sexual assault and threats related to sexual integrity decreased after 1999, while sexual torture involving humiliating and degrading acts against sexual integrity remained similar.

Table 3 shows the clinical impacts and Table 4 the physical, familial and social impacts, comparing the subsample who experienced some form of degrading treatment, ill treatment, or torture of a sexual nature (STS) versus those who suffered other forms of torture (non-sexual torture—NSTS).

Regarding clinical impacts, higher values were observed for PTSD (38.54 vs. 30.10, *p* < 0.01) and depression (14.93 vs. 11.29, *p* < 0.01) among STSs, indicating an increase in symptoms for both clinical conditions. In the medical area, a higher frequency of psychosomatic problems was reported in all items. These differences are more pronounced in the case of gastralgia, digestion problems, colon problems, erection difficulty and anorgasmia.

Concerning the impact of sexual torture on family dynamics, STSs showed a higher frequency across all types of family impacts. The difference was greater in the case of those who perceived themselves to have a diminished role within the family authoritative hierarchy and had difficulties adapting within the family, feelings of guilt towards their own families, feelings of inadequacy in their role as caregivers and decreased sexual desire towards their partner.

In terms of social and community impacts, STSs also showed a higher frequency across all types of impacts. The differences were more pronounced for those who reported feeling unsafe within the community, a loss of self-confidence or trust in others, and difficulties in adapting to society.

### 3.2. Evolution over Time

The long-term effects of sexual torture were compared by defining seven years after detention as the turning point (Table 5 and Table 6).

However, it is important to note that there was a decrease in clinical impact in terms of PTSD (41.51 vs. 35.88, *p* < 0.026). Similarly, there was a reduction in depressive symptoms (15.95 vs. 14.03 *p* < 0.71).

In terms of medical effects, some impacts show an increase in frequency over time, such as gastralgia ulcers, digestive problems or pain, colon issues, erectile difficulties, early or painful ejaculation, and anorgasmia, with the increase being particularly significant in most of these cases. Additionally, a decrease is observed in other impacts, such as chronic headaches and excessive weight loss.

In terms of family impacts, no major differences are observed over time. Most impacts remain consistent, with a slight increase in the frequency of perceiving a diminished role within the family hierarchy, difficulty adapting within the family, guilt towards the family due to the arrest, and feelings of alienation or difficulty relating to the spouse. A slight decrease is observed in the frequency of feeling like a disappointment to the family or loved ones, as well as feelings of alienation and difficulty relating to the spouse.

Regarding social and community impacts, these also remain relatively stable over time, with a slight increase in the frequency of loss of self-esteem or social status, and a slight decrease in the others.

## 4. Discussion

The prevalence and characteristics of different forms of ill treatment and sexual torture have been studied in a sample of Palestinian ex-detainees. This is the first published epidemiological study on the prevalence of sexual torture in men in which all respondents were specifically and systematically asked about this issue.

The results show that 82.6% of Palestinian male prisoners in our sample were subjected to various forms of sexual-related ill treatment or torture, including humiliation and degradation. The high frequency reported suggests that this is a pervasive practice.

Moreover, the data shows that these practices have persisted since 1987 and are not related to a specific period. The data also indicates that the prevalence of physical sexual torture is relatively low and that no cases of rape have been reported in this sample. After 1999, we observe a modest overall reduction in reported methods of sexual torture. Specifically, the incidence of physical sexual assaults and threats to sexual integrity declined. However, the prevalence of sexual torture characterized by humiliating and degrading acts remained similar, indicating that while overt physical tactics may have lessened, other insidious forms of psychological and sexual degradation persisted. An important cause for concern is that many of these men are minors. Our data corroborates the testimonial data of previous reports [34,40].

While the PCATi [2] study reported a prevalence of sexual torture of 4% in their sample, our study shows a prevalence of 60%. Potential explanations for this discrepancy include the following: (a) Interviews conducted by PCATi lawyers were administered while the detainee was still in prison or jail and the interview was recorded by the detention center authorities. This may have reduced the likelihood of the detainee testifying to these facts. In contrast, the interviews in this study were conducted privately at the victim’s home, within a trusted environment, where elements associated with guilt and shame were more likely to be revealed, and were conducted by psychologists with specializing in trauma. (b) Furthermore, these are interviews in which the victim’s spontaneous account is collected. In the PCATi study, there was no semi-structured interview format including systematic questioning on sexual-related torture and its consequences. (c) There are specific difficulties in expressing experiences of sexual torture among men in the Islamic context [13,17]. The context of continuous support from the TRC to the interviewees and the cultural proximity may have favored the openness to access these events.

The most frequent type of sexual torture was the use of humiliating and degrading acts against sexual integrity, reported by 68.1% of detainees. These findings support the notion that the use of humiliation as a form of psychological torture is a key element in the conflict, as evidenced by other studies [52] In total, 60.9% reported being submitted to forced nudity, 58% were beaten on their sexual organs, 35% were teased about their virility, 10% were threatened with rape or sexual assault and 9% were threatened with rape of female family members, among others. There are no reports of rape of detainees. These are forms of sexual aggression against men identified in other contexts of armed conflict [17,52].

The humiliating and degrading acts of a sexual nature may seem not to be as cruel as physical violence or rape [23]. Nevertheless, they have a significant impact on the psychological and physical level, as well as on the social and family dimensions [4,53].

In line with previous studies [18], in our study, there are higher scores on post-traumatic (STSs = 38.54; NSTSs = 30.1) and depressive (STSs = 14.93; NSTSs = 11.29) symptomatology in men who suffered sexual torture compared to those who had not.

The study also shows that there are also family and social impacts related to and/or exacerbated by sexual torture. These include a greater loss of confidence in oneself or others, a greater inability to adapt to society and to adapt within the family, a greater feeling of guilt towards their family, and a diminished role within the family hierarchy [13,24]. Furthermore, sexual functioning is affected, resulting in the lack of desire for sexual relations from one’s partner, which in turn affects the couple’s relationship. These findings are also consistent with previous studies [13,24,52].

Moreover, these impacts persist over time. When comparing the impact presented by people after 7 years or more, there is a decrease in clinical symptoms of post-traumatic stress or depression, although figures remain remarkably high. While many people experience acute post-traumatic stress symptoms following a traumatic event, not all develop long-term PTSD. Clinical definitions (e.g., DSM-IV/V) classify PTSD as ‘chronic’ if symptoms persist beyond three months, although this criterion has been criticized due to spontaneous recovery often occurring within the first 24 months without intervention. Longitudinal studies show a decline in PTSD prevalence, with one systematic review reporting a drop from ~28% at 1 month to ~17% at 12 months post trauma. A meta-analysis of long-term studies found that only 44% of individuals with PTSD experienced natural remission over ~3.5 years, suggesting the remainder had persistent symptoms. While the literature suggests that PTSD is considered ‘long-term’ after 3.5 years, we opted for a more restrictive criterion and defined it as seven years, double the typical threshold for ‘chronic’ PTSD [54,55,56].

Concurrently, the consequences on the social and familial environment persist over time and even intensify in instances where there is a loss of self-esteem and social standing or a sense of insecurity within the community. Age should also be considered when understanding these findings as possible influencing factors, as well as other issues related to factors associated with living in a context of conflict, insecurity, or other detentions. The impact that the therapeutic support received by the individuals in this study may have had on the improvement of their sequelae should also be considered.

These findings underscore the imperative for a more expansive conceptualization of sexual torture and its ramifications. It is essential to comprehend the full spectrum of sexual aggressions, including forced nudity, sexual humiliation, and witnessing non-consensual sexual acts. These acts, which were quite often not classified as torture, but as degrading treatment, produce severe suffering and damage that persist over time [18].

Despite numerous calls from international bodies (e.g., B’Tselem, PCATI, Human Rights Watch) for comprehensive investigations and accountability, there remains a persistent lack of transparency and willingness from the Israeli authorities to address these serious allegations Particularly, concerns regarding instances of sexual torture and mistreatment of Palestinian detainees continue to persist [1,2]. Such reluctance not only erodes public trust in the justice system but also perpetuates a vicious cycle of abuse and impunity. Furthermore, the long-term psychological and physical impact on the victims is profound; indeed, it has been observed to leave long-lasting scars, which in turn contribute to a broader climate of fear and oppression within the Palestinian community. It is thus clear that the issue must be addressed by a concerted effort to ensure that accusations of this nature are investigated with impartiality and that survivors receive the justice and reparations that they deserve according to international law.

### Limitations

The data pertains solely to male Palestinian detainees, which may limit the generalizability of the findings to other groups, including female detainees or detainees of different origins and cultural backgrounds. Although the sample in this article is robust, the relatively small number of detainees included, given the high number of detentions during the studied period, makes it limited in comparison to the scale of the issue. In this regard, for future epidemiological studies, it would be valuable to use confidence intervals calculated for the estimated prevalence.

Data on the long-term effects of sexual torture, using a seven-year post-detention benchmark, must be interpreted with caution. Many participants experienced subsequent re-arrests or additional human rights abuses, preventing a clear causal link between the index detention and outcomes measured seven years later and precluding a uniform “post-release” window across respondents. Interview timing relative to release was highly variable, and any time-dependent association should therefore be viewed with caution. Moreover, some medical impacts may also reflect the effects of time and aging rather than detention alone.

It is essential to conduct studies with improved controls to enhance the understanding of the findings and employ methods for controlling covariates that may have been overlooked in this study, such as detention duration, the cumulative impacts of multiple detentions, and other factors not considered here. Secondly, the retrospective nature of the data may introduce challenges related to the accuracy and consistency of reporting over time. Additionally, the sensitive and traumatic nature of the subject could have led to under-reporting or variations in the accounts of torture, further complicating the analysis.

## 5. Conclusions

Our data shows a high prevalence of the use of degrading treatment, ill treatment, and sexual torture of Palestinian male prisoners by Israeli state security forces. While rape has not been reported, there are other types of techniques related to dignity and identity, in line with previous studies [10,13,14].

As this is not a random sample but comes from an existing database, originally compiled over time among former detainees, the data presented in this study does not provide definitive data on the prevalence of sexual ill treatment and torture in former political prisoners. Nevertheless, the accounts provide a compelling indication that sexual torture may have been a practice employed by Israeli security forces. There are grounds to suspect that it is still practiced.

Furthermore, the data shows that sexual torture has profound and long-term medical, psychological, social, and familial repercussions. These allegations of sexual torture are not investigated by the Israeli authorities, despite their adherence to international and national conventions concerning sexual violence. Furthermore, victims do not receive compensation for the damage caused by the sexual torture they have suffered [2].

The eradication of sexual torture requires a coordinated effort between national and international actors to ensure justice, reparation, and effective prevention mechanisms. In light of this data, the Israeli state authorities must allow independent investigations and conduct internal investigative processes to stop these practices, with the participation of international organizations such as the United Nations. These investigations must ensure the prosecution of those responsible and hold perpetrators accountable at all levels. Additionally, it is essential to establish monitoring mechanisms in detention centers and develop secure and confidential channels for reporting torture and sexual violence. Furthermore, victims must be guaranteed comprehensive reparation and adequate compensation for the harm suffered.

## Figures and Tables

**Table 1:** Epidemiology of sexual torture among male Palestinian detainees (N = 517).

	Any Type of Sexual Torture (%)	Threats Related to Sexual Integrity (%)	Humiliating and Degrading Acts Against Sexual Integrity (%)	Physical Sexual Assault—Attacks Involving Sexual Violence (%)
Total (n = 517) Age of detention	82.6	14.7	68.1	59
<18 years (n = 179)	84.9	13.4	67	59.2
18–24 years (n = 258)	80.6	15.1	69.8	57.4
>25 years (n = 80)	83.6	16.3	65	63.7
Region of detention				
West Bank (n = 400)	83.5	15.3	69.8	57 *
Gaza (n = 117)	81.3	13.1	62.6	68.2 *
Year of detention				
Before 1999 (n = 310)	84.5	18.4 ***	67.4	65.2 ***
After 1999 (n = 207)	79.7	9.2 ***	69.1	49.8 ***

Chi-Square test: * = *p* < 0.05; *** = *p* < 0.001.

**Table 2:** Types of sexual torture among male Palestinian detainees and differences over time (n = 517).

Type of Sexual Torture	%		
**Threats related to sexual integrity—threats of sexual harm**	Total	Before 1999	After 1999
Projecting sounds similar to household members being exposed to rape	3.1	2.5	0.6
Threats to rape female family members	9.1	12.3 **	4.3 **
Threats to sexually assault or rape the detainee	9.9	11.6	7.2
**Humiliating and degrading acts against sexual integrity**			
Having to undress	60.9	61	60.9
Making fun of the detainee’s body while they are undressed	28.2	30	25.6
Making fun of detainees’ manhood	35.6	37.1	33.3
Bringing in partially undressed female soldiers to provoke handcuffed inmates and cause psychological breakdown	7	8.1	5.3
**Physical sexual assault—attacks involving sexual violence**			
Beating sexual organs while the detainee is partially undressed	58	64.5 ***	48.3 ***
Sexual assaults	2.9	2.6	2.9

Chi-Square test: ** = *p* < 0.01; *** = *p* < 0.001.

**Table 3:** Comparison of clinical impacts in sexual torture and non-sexual torture.

Type of Impact	Type of Torture	N	Mean	SD	T	DF
**Clinical Impact**						
PTSD (PCL-5)	NSTSs	61	30.10	21.06	−2.83 **	88.31
	STSs	258	38.54	20.39		
Depression (Beck)	NSTSs	115	11.29	10.76	−3.20 **	182.61
	STSs	400	14.93	10.61		

NSTs = non-sexual torture survivors; STSs = sexual torture survivors. *t*-test: ** = *p* < *0*.01.

**Table 4:** Comparison of medical, familial and social impacts in sexual torture and non-sexual torture.

Type of Impact	Type of Torture				
**Medical Impact**	**NSTSs**	**STSs**	**Chi-2**	**Sig.**	**Effect Size**
Gastralgia/gastric ulcer	17.8	34	9.151	0.002	0.132
Problems or pains in digestion	12.2	22.3	4.624	0.032	0.094
Problems in the colon	8.9	20.2	6.367	0.012	0.110
Chronic head pains	18.9	24.7	1.390	0.148	0.052
Excessive loss of weight	12.2	15.3	0.557	0.455	0.033
Erectile difficulties	0	7.5	7.111	0.008	0.117
Early or painful ejaculation	5	15	2.930	0.087	0.104
Anorgasmia	2.6	16.8	5.381	0.020	0.141
**Family** **Impact**					
Diminished role within the family hierarchy	5.6	17.2	7.638	0.006	0.121
Difficulty adapting within the family	7.9	18.1	5.658	0.017	0.104
Guilt towards family due to the arrest	5.6	18.6	9.055	0.003	0.132
Feelings of causing disappointment to the family or loved ones	14.5	25.4	4.773	0.029	0.096
Feelings of alienation and difficulty relating to your spouse	2.2	6.1	2.17	0.141	0.065
Lack of desire for intercourse with your spouse	0	23.1	5.998	0.014	0.214
**Social impact**					
Loss of self-esteem or social status	11.2	14.8	0.777	0.378	0.039
Difficulty adapting to friendships	11.2	19.3	3.252	0.071	0.079
Feeling unsafe within the community	33.7	59.4	19.668	0.00	0.192
Loss of self-confidence or trust in others	13.5	27.7	7.900	0.005	0.123
Difficulty adapting to society	10.1	25.9	10.348	0.001	0.141

NSTSs = non-sexual torture survivors; STSs = sexual torture survivors.

**Table 5:** Comparison of clinical impacts in sexual torture over time.

Type of Impact	Type of Torture	N	Mean	SD	T	DF
**Clinical Impact**						
PTSD (PCL-5)	MTI	122	41.51	20.47	2.23 *	256
	LTI	136	35.88	19.87		
Depression (Beck)	MTI	187	15.95	11.07	1.81	398
	LTI	213	14.03	10.12		

LTI = long-term impact (sexual torture more than 7 years ago); MTI = medium-term impact (sexual torture less than 7 years ago). *t*-test: * = *p* < 0.05.

**Table 6:** Comparison of medical, familial and social impacts in survivors of sexual torture over time.

Type of Impact	Type of Torture				
**Medical Impact**	**MTI**	**LTI**	**Chi-2**	**Sig.**	**Effect Size**
Gastralgia/gastric ulcer	24.5	37.8	10.669	0.001	0.142
Problems or pains in digestion	11.7	29.3	24.677	0.000	0.214
Problems in the colon	12.1	24.3	13.019	0.000	0.157
Chronic head pains	23.8	23.6	0.005	0.941	0.003
Excessive loss of weight	15.2	14.3	0.092	0.762	0.013
Erectile difficulties	2.7	9.7	10.871	0.001	0.144
Early or painful ejaculation	6.6	16.4	4.455	0.035	0.129
Anorgasmia	11.8	16	0.728	0.394	0.052
**Family** **Impact**					
Diminished role within the family hierarchy	14.5	15.9	0.207	0.650	0.02
Difficulty adapting within the family	14.5	18.2	1.332	0.249	0.051
Guilt towards your family due to the arrest	14.8	17.8	0.838	0.36	0.04
Feelings of causing disappointment to the family or loved ones	26.2	23.9		0.342	
Feelings of alienation and difficulty relating to your spouse	2.7	8.1	7.344	0.007	0.118
Lack of desire for intercourse with your spouse	27.8	17.8	0.997	0.318	0.089
**Social Impact**					
Loss of self-esteem or social status	13.7	14.7	0.118	0.731	0.015
Difficulty adapting to friendships	19.5	16.3	0.925	0.336	0.042
Feeling unsafe within the community	55.1	54.9	0.002	0.961	0.002
Loss of self-confidence or trust in others	27.1	23.4	0.888	0.346	0.042
Difficulty adapting to society	23.9	22.5	0.149	0.699	0.017

LTI = long-term impact (sexual torture more than 7 years ago); MTI = medium-term impact (sexual torture less than 7 years ago).

## Data Availability

The raw data supporting the conclusions of this article will be made available by the authors on request.
